# Antibiotics versus biofilm: an emerging battleground in microbial communities

**DOI:** 10.1186/s13756-019-0533-3

**Published:** 2019-05-16

**Authors:** Divakar Sharma, Lama Misba, Asad U. Khan

**Affiliations:** Medical Microbiology and Molecular Biology Laboratory, Interdisciplinary Biotechnology Unit, Aligarh Muslim University, Aligarh, 202002 India

**Keywords:** Biofilm, Antibiotics resistance, Drug resistance bacterial infection

## Abstract

Biofilm is a complex structure of microbiome having different bacterial colonies or single type of cells in a group; adhere to the surface. These cells are embedded in extracellular polymeric substances, a matrix which is generally composed of eDNA, proteins and polysaccharides, showed high resistance to antibiotics. It is one of the major causes of infection persistence especially in nosocomial settings through indwelling devices. Quorum sensing plays an important role in regulating the biofilm formation. There are many approaches being used to control infections by suppressing its formation but CRISPR-CAS (gene editing technique) and photo dynamic therapy (PDT) are proposed to be used as therapeutic approaches to subside bacterial biofim infections, especially caused by deadly drug resistant bad bugs.

## Introduction

Bacterial biofilms are serious global health concern due to their abilities to tolerate antibiotics, host defence systems and other external stresses; therefore it contributes to persistent chronic infections [27, 33]. Biofilms are immobile microbial communities which colonize and grow on surfaces of medical implants such as sutures, catheters and dental implants, by self-produced extracellular polymeric substances and cause infections which can only be treated by their removal and leads to the unaffordable treatment as well as mental-illness to patients [26, 52]. Biofilm comprises of the crammed bacterial population by extra-cellular matrix (ECM) which possesses bacterial secreted polymers such as exopolysaccharides (EPS), extracellular DNA (e-DNA), proteins and amyloidogenic proteins [140, 146]. Formation of biofilm is well-regulated multi step events such as (i) adsorption of molecules (macro and micro molecules) to surfaces; (ii) bacterial adhesion to the surface and release of extracellular polymeric substances (EPS); (iii) colony formation and biofilm maturation. Metabolic activity of the bacterial biofilm communities have altered as compared to the planktonic one such as increased rates of EPS production, activation or inhibition of particular genes associated with biofilm formation and decreased growth rate [39].

Biofilms provides the protection to the microorganism not only from altered pH, osmolarity, nutrients scarcity, mechanical and shear forces [28, 41, 82] but also block the access of bacterial biofilm communities from antibiotics and host’s immune cells [27, 126]. Therefore, biofilm matrix gives the additional resistance power to bacteria which makes them to not only tolerate harsh conditions but also resistant to antibiotics which lead to the emergence of bad bugs infections like multi drug resistant, extensively drug resistant and totally drug resistant bacteria.

Formation of biofilms in mycobacteria can be defined as similar as other biofilms communities. However, some mycobacteria can develop biofilms on surfaces as well as the air-media interface [100]. Due to deficiency of surface fimbriae or pili in mycobacteria, few proteins have been reported as potential factors for the aggregation and attachment of mycobacteria cells [83]. Mycobacteria do not produce the usual exopolysaccharide but it has ability to attach a varieties of surfaces [150] and form fully developed biofilms [101, 149].

Panels of studies have reported the role of various molecules in the biofilm formation and maintenance of its composition. Glycopeptidolipids are indispensable for initial surface attachment during biofilm formation in *Mycobacterium smegmatis* [111]. It has been suggested earlier that shorter chain mycolic acids may form a hydrophobic extracellular matrix [102]. Shorter-chain mycolic acids have essential in the development of biofilm structure of the non-motile mycobacteria [100]. Mycolic acids, a potential permeability barrier could be associated to the higher resistance to antibiotics [149]. These molecules also have an essential role in sliding motility that can be correlated with biofilm spreading [81, 111, 112]. Role of GroEL1 chaperone in the biofilms development of *M. smegmatis* has already been reported [35, 101, 102].

Growth pattern of Mycobacterium species are different such as *M. chelonae* and *M. fortuitum* form biofilm as vertical and entire surface growth, respectively [93]. Cording has been associated with pathogenicity of various mycobacterial species [46, 81, 93, 145] and its link in the biofilm development by *M. tuberculosis* are underway [22].

Mycobacterial species form biofilms in the various environmental reservoirs [37, 38, 115, 116] and these reservoirs participate in the outbreaks of mycobacterial infections [59, 133, 135, 136]. In one of the studies, *M. chimaera* (nosocomial pathogen) biofilm has shown to be associated with their contamination of heater-cooler units of surgery [62].

Discovery of biofilms in *M. tuberculosis* suggested that the infection associated with clinical biomaterial and prosthetic joints in particular [16, 45, 123] and removal of these biomaterials was indispensable to manage these infections otherwise it could leads to the development of antibiotic resistance phenomenon. It was reported in previous study that *M. tuberculosis* can also develop a biofilm in vitro [100]. It was suggested that mycolic acids as well as DNA, were crucial in the development and regulation of *M. tuberculosis* biofilms [94, 101].

Few studies suggest that casseous necrosis and cavitation in lung tissue of *M. tuberculosis* is potentially due to the biofilms formation [13, 67] which might decrease in the activity of anti-mycobacterial drugs against *M. tuberculosis* biofilms [55, 100]. These discoveries open the new research areas which not only explore the mechanism of biofilms formation but also the antibiotics resistance and these potential targets could help in the development of alternative therapies against drug resistance tuberculosis.

### Ultrastructure of biofilm

Microbial biofilm is the grouping of sessile microbial communities which is attached with substratum and embedded in the self produced pool of non-crystalline extracellular polymeric matrix [52]. Bacterial biofilm communities differ from the planktonic ones in different ways such as growth rate, gene expression, transcription and translation because these biofilms communities live in different microenvironments which have higher osmolarity, nutrient scarcity and higher cell density of heterogeneous bacterial communities. Formation of the three-dimensional structure of biofilm is the dynamic process by heterogeneous bacterial communities. Bacteria living within the biofilms are protected from the varieties of environmental stresses, such as desiccation, antimicrobials attack by the immune system and ingestion by protozoa hence this architecture makes the biofilm communities to advance as compared to planktonic one [143]. Coordination within the biofilm via cell-to-cell communication called quorum sensing (QS) in which accumulation of signaling molecules in extracellular environment leads to regulation of the specific genes expression. Some bacterial species use QS to coordinate the disassembly of the biofilm community [121].

Development of biofilms is multi step process. It starts with the initial adherence of bacteria to the substratum and irreversible attachment followed by their colonization in which modification in genes/proteins expression occurs followed by exponential growth phase. The exopolysaccharides (EPS) and water channels formation occur, facilitating nutrient supply which leads to the maturation of the biofilms. Ultimately surface detachment of the cells starts in the environments which again restart/recycle the biofilm formation onto the new surfaces.

### Infections associated with biofilms

Approximately 80% of chronic and recurrent microbial infections in the human body are due to bacterial biofilm. Microbial cells within biofilms have shown 10–1000 times more antibiotics resistance than the planktonic cells [79]. Biofilm is formed in diverse environmental niches, including freshwater rivers, rocks, deep-sea vents and hydrothermal hot springs. Biofilm-related infections can be broadly divided into two types. The biofilms may be formed on the abiotic surfaces, especially infections associated with indwelling medical devices [34] and native biofilm infections of host tissue [21]. Urinary tract and bloodstream infections can be caused by the biofilm initially formed on medical implants, such as heart valves, catheters, contact lenses, joint prostheses, intrauterine devices and dental unit. These infections can only be treated by removal of the implants which not only increasing the cost of the treatment but also it becomes problematic for patients [26]. Host tissue related biofilm infections are often chronic, including chronic lung infections of cystic fibrosis patients, chronic osteomyelitis, chronic prostatitis, chronic rhinosinusitis, chronic otitis media, chronic wounds, recurrent urinary tract infection, endocarditis, periodontitis and dental caries [21]. Some of the major biofilm associated infections causing human diseases are listed in Table 1.

**Table 1:** Bacterial species involved in biofilm associated infection and their adherent surfaces

S. No.	Bacterial Species	Infection/Diseases	Surface	References
1	*Streptococcus mutans*	Dental caries Endocarditis	Tooth surface Vascular grafts	[1, 84]
2	*Enterococcus faecalis*	Endocarditis Root canal infection	Heart valves Urinary catheters Tooth Central venous catheters	[85]
3	*Klebsiella pneumonia*	Pneumonia Respiratory tract infection Urinary tract infection Pyogenic liver abscess	Lungs Liver	[24]
4	*Pseudomonas aeruginosa*	Nosocomial infection Otitis media Cystic fibrosis	Central venous Catheters Middle ear Prostheses Lungs Contact lenses	[53, 139, 142]
5	*Staphylococcus sp (Staphylococcus aureus; Staphylococcus epidermidis).*	Nosocomial infections Chronic wounds Endocarditis Mucoloskeletal Infections Otitis media	Sutures Central venous catheters Arteriovenous shunts Prostheses Surfaces/deep skin Prostheses, Heart valves Bones, Middle ear	[6, 109]
7	*Escherichia coli*	Bacterial prostatitis Urinary tract infection Otitis media	Prostheses, Urinary tract Urinary catheters Middle ear	[56]
8	*Haemophilus influenza*	Otitis media	Middle ear	[113, 130]
9	*Burkholderia cepacia*	Cystic fibrosis	Lungs	[152]
10	*Mycobacterium tuberculosis*	Tuberculosis	Lungs	[110]

### Resistance to antibiotics in biofilms communities

**Fig. 1:**
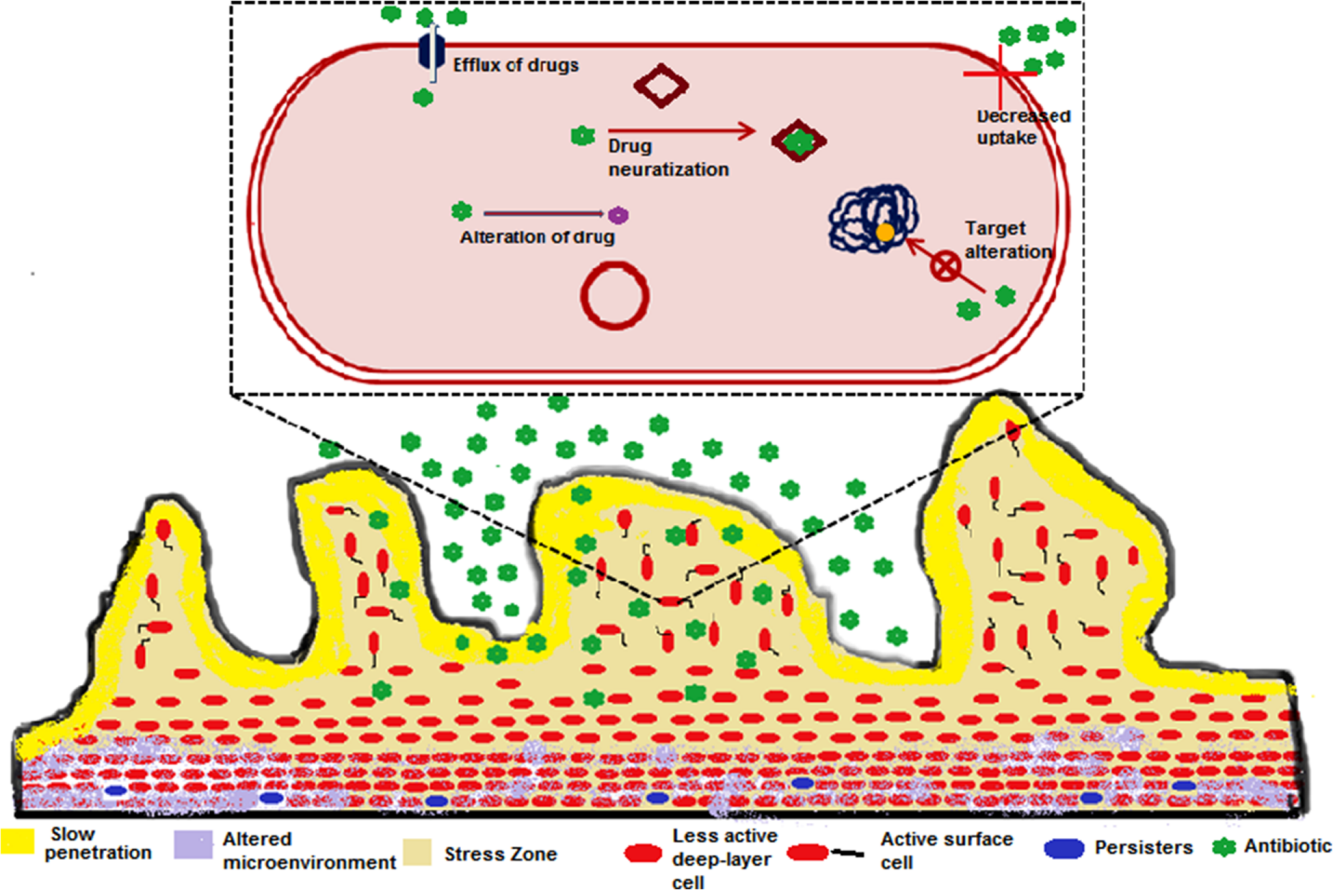
Diagrammatic representation of the potential mechanisms of antibiotic resistance in biofilms communities

Antibiotic resistance of bacteria in the biofilm communities contributes to the chronic infections. Resistance mechanisms of biofilm communities are not similar as the planktonic ones such as target site mutations, lower cell permeability, efflux pumps, drug modifying enzymes and drug neutralizing proteins [14, 70, 72, 76, 77, 96, 117, 118, 137, 138]. A panel of studies suggested that conventional antibiotic resistance mechanisms are unable to explain the various cases of antibiotic-resistant biofilm infections [2, 5, 19, 144]. On the basis of these studies we can’t ignore the possibility of the conventional resistance mechanisms in biofilms which contribute to the antibiotic resistance. It has been reported earlier that repeated exposure of ceftazidime in biofilm-growing *Pseudomonas aeruginosa* developed the conventional type of intrinsic antibiotic resistance in biofilms infections [10]. In biofilm communities, antibiotics resistance appears due to various strategies (Fig. 1) such as slow or incomplete penetration of the antibiotics into the biofilm [42, 71, 78, 95, 119, 124, 125], an altered chemical microenvironment within the biofilm [32, 108, 129, 132, 147, 151] and a subpopulation of micro-organisms in a biofilm {a type of cell differentiation like to spore formation} [25, 29, 43]. These mechanisms are the consequences of the multicellular nature of biofilms which leads to the antibiotics resistance of biofilm communities [30] along with the known conventional resistance mechanisms and makes the failure of treatment strategy. Multicellular nature of biofilm is the key factor of antibiotics resistance of biofilm communities which is the actual cause of the resistance mechanisms as discussed above. A series of researches exists on formation of biofilm as a multicellular developmental process [65, 98, 127]. Extracellular polymeric substances (EPS) hold the bacterial cells together and lead to the development of multicellular consortia which makes the heterogenous environment inside the biofilm and initiates the biofilm to function as a multicellular system. Biofilm development is well organized and during its development intercellular and intracellular signaling occurs. A panel of genes/proteins are upregulated as well as downregulated for attachments of bacteria onto substratum surface and pathways differentiation [15, 27, 98, 99, 141]. Maturation of the biofilm into complex structures is regulated by the signalling among the cells by the quorum sensing process [31]. Multicellularity nature of biofilm bacterial communities is responsible for antibiotics resistance; if we can disrupt any step in the formation of multicellular structure of the biofilm than antibiotics efficacy as well as the host defences might be increased which leads to quick treatment of this persistent infection. On the basis of these observations we can say that multicellular developmental process of the biofilms are important because its insight will open up new targets and approaches for designing new drug molecules against antibiotics resistant microorganisms.

Antibiotics resistant state of the biofilm cells lead to a treatment complications in the series of human infections which include biofilm formation on various biological implants such as, heart catheters, urinary catheters, joint implants and replacement of heart valves [120]. Biofilms pose a threat to the human race because of their persistent nature and plays a major role in certain pathogenic infections [40, 58, 104, 120]. Studies suggested that role of EPS have been conferring tolerance to aminoglycosides [41, 61]. EPS might quench the activity of antibiotics that diffuse through the biofilms via diffusion–reaction inhibition phenomenon, which may chelates the antibiotics by complex formation or degrade through enzymatically based reactions [17, 105]. Stationary phase (a slow or non-growth phase of the bacterial life cycle) and viable-but-nonculturable state (VBNC state or a state of dormancy) are the ways of survival for bacterial biofilms communities under antibiotics stress [20, 73]. Biofilms possesses many cells of stationary phase which have the decreased antibiotics susceptibility to the antibiotics. Among them at least 1% becomes tolerant to antibiotics [4, 80]. Many biofilms communities enter into the stationary phase with time which suggested that older biofilms show higher tolerance to antibiotics [89]. Persisters are another dormancy state of bacterial subpopulation, which have the multidrug tolerance phenotypic rather than genetic variations [8, 47]. In stationary state of biofilms communities, persisters might be the prevalent [60].

One of the antibiotics resistance mechanisms of biofilms communities is the uptake of resistance genes by horizontal gene transfer [79]. Biofilms provides the compatible conditions for the horizontal gene transfer such as high cell density, increased genetic competence and accumulation of genetic elements or uptake of resistance genes [41]. Conjugation is the only mechanism of horizontal transfer of resistant genes in biofilms and may confirm the resistance to several antibiotics. Few studies suggested that conjugation has been shown more efficient in biofilms as compared to planktonic ones [66, 75, 114, 134].

A penal of studies reported that in vitro mycobacterial biofilms were resistant to antibiotics (amikacin and clarithromycin) or disinfectants [44, 103]. It has been reported earlier that differences between the MIC and minimum biofilm eradication concentration (MBEC) in 4 species of RGM [91] and suggested that ciprofloxacin as an effective antibiotic against these biofilms as compared to clarithromycin and amikacin.

Few studies shown the effect of antibiotics in different stages of biofilm development [90–92] and revealed that at early stage of biofilm development antibiotics treatment was more effective, probably due to the cells which are not completely adapted into biofilm communities. In an attempt to evaluate mechanisms for these resistance patterns, it has been suggested that permeability of anti-tuberculosis drugs were independent among the mycobacterial species [103]. Metabolic state and activation of resistance genes (like methylases) are indispensable for the development of antibiotics resistance in mycobacteria [36, 44].

### Alternative approaches to control the biofilm related infections

**Fig. 2:**
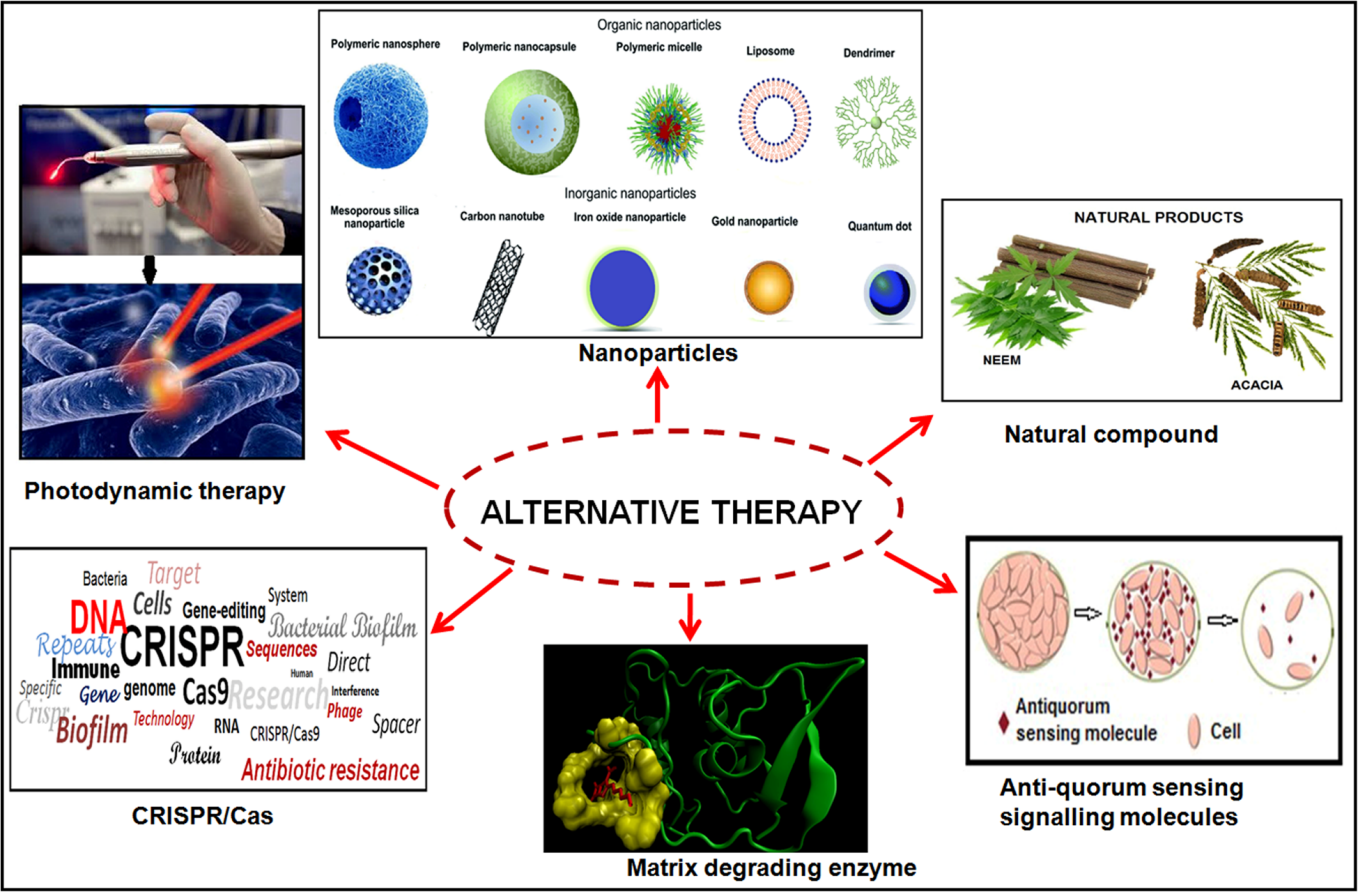
Diagrammatic representation of the alternative approaches against antibiotic resistant biofilms communities

Successful treatment of biofilm-associated infections is troubled due to high antibiotic resistance in these bacterial communities. Classical antibiotics chemotherapy is unable to completely eradicate bacterial cells which are situated in the central region of the biofilm and leads to the emergence of the worsen situation globally. Therefore to overcome the drug resistance of bacterial biofilm communities; alternative strategies (Fig. 2) and novel antibiofilm agents have been studied earlier [9, 48–50, 54, 63, 64, 69, 74, 86–88, 92, 128, 131, 148, 153, 154].

Naturally produced small molecules by bacterial biofilm communities such as D-amino acids and Polyamine norspermidine; induced the dispersal of mature biofilms which could prevent biofilm formation in *S. aureus* and *E. coli* [50, 63, 64]. These molecules could be used as antibiofilm agent in the biofilm dispersal strategy. Muñoz-Egea et al. [92] reported that antibiofilm molecules (N-acetylcysteine/NAC and Tween 80) alone and in combination with antibiotics were effective against non-pigmented RGM biofilms [92]. Tween 80 is more active against mycobacterial biofilm than NAC because mycobacterial cell wall as well as extracellular matrix possesses high lipid content and suggested that synergistic effect of drugs and anti-biofilm agent may effective in the treatment of infections associated with mycobacterial biofilms communities.

Degradation of the biofilm matrix by biofilm matrix degrading enzymes (DNase I, Dispersin B (DspB) and a-amylase) is also another promising antibiofilm strategy. Degradation of biofilm structural component allows the increased penetration of antibiotics which enhances the antibiotics effeciency. DNase I, DspB and α-amylase degrade eDNA, biofilm matrix and exopolysaccharides, respectively [128, 131], which not only inhibit biofim formation but also degrading the mature biofilms in various microbes such as *S. aureus*, *Vibrio cholerae* and *P. aeruginosa* [57].

Formation of biofilms were controlled by the quorum sensing (QS) signalling genes and their products. Various inhibitors/compounds are able to disturb the QS signalling cascade and used as alternative therapy for the treatment of biofilm-related infections. Halogenated furanone isolated from *Delisea pulchra* (marine algae) interrupt the bacterial QS signalling [74]. Recently, Kaur et al. [58] reported an acyclic diamine (ADM 3), showed better antimicrobial activity and antibiofilm activity [58]. Attenuation of bacterial QS signalling by ginseng extract, garlic extract, usnic acid and azithromycin possesses inhibitory activity against bacterial and fungal biofilms [18, 51, 122]. Signalling molecule nitric oxide (NO) disperse the biofilms in *P. aeruginosa* and enhances the activity of antimicrobial compounds via stimulation of c-di-GMP-degrading phosphodiesterases, which induce a switch to planktonic growth [12]. Most recently our group used the CRISPRi technology to knockdown the luxS gene of QS signalling and fimbriae associated gene (fimH) for controlling the biofilm mediated infections [153, 154].

Bacterial and actinomycetes have been shown to produce bioactive agents/natural compounds with antibiofilm properties. Methanolic extract of a coral-associated actinomycete helps to reduce biofilm formation of *S. aureus* [11]. Another natural product, 4-phenylbutanoic acid show high antibiofilm activity against Gram positive and Gram negative bacteria [97]. Azadiracta *indica* (Neem) and Acacia extracts showed antimicrobial effect against *S.mutans* and *S. faecalis* [3, 7].

Nanoparticles have considered as the alternative of the antibiotics to combat multidrug resistance and biofilm based infections (Pelgrift & Friedman [106]). Limitations of the conventional antibiotic treatments (reduced penetration and retention in cell or biofilm) were overcome by their nano-formulations which have the ability to cross the biological barrier. Since the last few years, different type of nanoparticles have been used as antimicrobial and antibiofilm metal nanoparticles, organic nanoparticles, green nanoparticles and their combinations [9]. A panel of reports exists on nanoparticles based elimination of bacterial biofilm communities (Hernández-Sierra et al. [48, 49, 54, 69]). Kulshrestha et al. [68] reported that suppressive effect of CaF2-NPs on genes associated with major virulence factors (*vicR, gtfC, ftf, spaP, comDE*) of *S. mutans* and suggested the suppression of enzymatic activity associated with glucan synthesis, cell adhesion, acid production, acid tolerance and quorum sensing which leads to biofilm inhibition [68]. In the last few years’ photodynamic therapy (PDT) was used to treat various type of infection like as bacterial, fungal, viral, protozoa or even parasitic infection. It has reported earlier that PDT has sufficiently reduced the clinically-relevant microbes, such as drug resistant Gram-positive and Gram-negative bacteria [23]. PDT has significant advantages over conventional treatment owing to its ability of selective binding to the membranes of pathogenic cells and the possibility for accurate delivery of light to the affected tissue for the maximal damage of microbes as well as minimal damage of the host [107]. Recently, our group has shown that PDT could be used to eliminate the biofilms related issues in *S.mutans* infection (Lama et al., 2016; 2017; 2018).

## Conclusion and future prospects

Bacterial antibiotic resistance is also one of the consequences of the bacterial biofilm communities which contribute to the chronic infections. These biofilm communities have few additional resistance mechanisms as compared to the planktonic ones which hamper the treatments option and leads to emergence as well as spreading of the chronic bad bugs. Emergence and spreading of multidrug resistant, extremely drug resistant and total drug resistant strains of *M. tuberculosis* have worsened the current situation across the globe. In this timeline review we have discussed the mechanisms of antibiotics resistance in biofilms communities and alternative therapeutic options to combat the resistance mediated by chronic bacterial biofilm infections. Alternative approaches, like nanoparticles based antibiotics formulation, novel anti-biofilm agents, CRISPRi gene editing technologies and photodynamic therapy might be the future options to treat the infections caused by multidrug resistant, extremely drug resistant and total drug resistant strains of *M.tuberculosis* which might be one of the ways to achieve the goal of TB free world declared by WHO.

## Abbreviations

ECM: Extra-cellular matrix
e-DNA: Extracellular DNA
EPS: Exopolysaccharides
MBEC: Minimum biofilm eradication concentration:
MIC: Minimum inhibitory concentration
PDT: Photodynamic therapy
QS: Quorum sensing
